# Temporal dynamics of immune effector cell‐associated haematotoxicity: Linking early and late cytopenias after chimeric antigen receptor T cells

**DOI:** 10.1111/bjh.70659

**Published:** 2026-07-10

**Authors:** Malvina Bichon, Roch Houot, Mana Alshehri, Guillaume Manson, Faustine Lhomme, Samuel Vic

**Affiliations:** ^1^ Univ Rennes, CHU Rennes, Service d'Hématologie Clinique et Thérapie Cellulaire Rennes France; ^2^ INSERM‐UMR 1236, Établissement Français du Sang Rennes France; ^3^ Advanced Therapeutic Medicinal Products Department, Biological Products Directorate, Executive Directorate of Quality Evaluation of Medicines, Drug Sector Saudi Food and Drug Authority Riyadh Saudi Arabia

**Keywords:** CAR T‐cells, cytopenia, haematotoxicity, ICAHT, lymphoma, multiple myeloma

To the Editor,

Chimeric antigen receptor (CAR) T‐cell therapy has transformed the treatment of B‐cell and plasma cell malignancies but carries a unique toxicity profile that includes cytokine release syndrome (CRS), immune effector cell‐associated neurotoxicity syndrome (ICANS) and immune effector cell‐associated haematotoxicity (ICAHT). Cytopenias represent the most frequent grade ≥3 adverse event regardless of CAR construct or disease, with grade ≥3 neutropenia, anaemia and thrombocytopenia reported in 72%–91%, 49%–69% and 49%–62% of patients respectively.^1^, ^2^, ^3^ Three reconstitution patterns have been described—quick recovery, intermittent recovery and aplastic phenotype—the latter being associated with both increased relapse and non‐relapse mortality.^1^, ^4^ The European Hematology Association/European Society for Blood and Marrow Transplantation (EHA/EBMT) consensus grading now incorporates timing (early, days 0–30; late, days 30–90), depth and duration of neutropenia, allowing standardized comparisons.^5^ Severe (grade 3–4) early ICAHT has been linked to prolonged hospitalization, transfusion dependence, severe infections and increased non‐relapse mortality.^5^ By contrast, late ICAHT remains poorly characterized, since most events occur in outpatients with heterogeneous monitoring and the relationship between early and late events has not been clearly established. Whether early ICAHT can predict late ICAHT carries direct implications for surveillance and supportive care. We addressed this question in a single‐centre retrospective cohort.

We retrospectively analysed all consecutive adult patients (≥18 years) treated with CAR T‐cells at CHU Rennes between 23 August 2018 and 31 July 2024 for B‐cell lymphoma or multiple myeloma (MM), with complete biological follow‐up (≥2 blood tests per month) for at least 90 days. Patients with acute lymphoblastic leukaemia, disease progression at 1 month or death within 3 months post‐infusion were excluded (Figure S1). Early ICAHT was graded using the validated automated algorithm of Liang et al^6^ applied to absolute neutrophil count (ANC) thresholds (501 or 101 cells/μL) with linear interpolation for up to 7 consecutive days of missing values; neutrophil recovery followed Center for International Bone and Marrow Transplant Research (CIBMTR) criteria. Late ICAHT was graded by EHA/EBMT criteria based on the two lowest ANC nadirs between days 30 and 90.^5^ CRS and ICANS were graded by American Society for Transplant and Cellular Therapy (ASTCT) consensus criteria.^7^ Granulocyte colony‐stimulating factor (G‐CSF) was administered for ANC <0.5 × 10^9^/L per institutional protocol. Categorical variables were compared by chi‐square or Fisher's exact test, and continuous variables by Kruskal–Wallis test; *p* < 0.05 was considered significant. Analyses were performed in R. All patients provided informed consent for the non‐interventional use of personal data.

Of 330 consecutive patients screened, 169 met inclusion criteria. Median age was 68 years (range 18–80); 65% were male; 10% had performance status ≥2. The majority had diffuse large B‐cell lymphoma (DLBCL, 55%), followed by MM (25%), follicular lymphoma (FL, 10%), mantle cell lymphoma (MCL, 6%) and other lymphomas (4%). Axicabtagene ciloleucel (axi‐cel) was the most frequently used product (51%), followed by idecabtagene vicleucel (ide‐cel) (25%). Median number of prior treatment lines was two (range 1–10); 33% had received prior autologous haematopoietic stem cell transplantation; 40% had a CAR‐HEMATOTOX score ≥2. Any‐grade CRS occurred in 90% (grade ≥3, 1%) and ICANS in 46% (grade ≥3, 20%). Baseline and post‐infusion characteristics are summarized in Table 1.

**TABLE 1:** Patients' baseline characteristics and post‐infusion toxicities.

Characteristic	All patients (*n* = 169)	Early ICAHT only (*n* = 38)	Early + late ICAHT (*n* = 102)	Late ICAHT only (*n* = 13)	*p* value
Age (years), median (range)	68 (18–80)	69 (24–79)	68 (18–80)	71 (55–80)	0.09
Male, *n* (%)	110 (65)	32 (84)	57 (56)	8 (62)	**0.005**
PS ≥2, *n* (%)	17 (10)	3 (8)	11 (11)	1 (8)	1.000
Disease, *n* (%)					0.08
DLBCL	93 (55)	19 (50)	65 (64)	5 (38)	
FL	16 (10)	3 (8)	4 (4)	4 (31)	
MCL	10 (6)	4 (11)	5 (5)	0	
Other lymphomas	7 (4)	3 (8)	4 (4)	0	
Multiple myeloma	43 (25)	9 (23)	24 (23)	4 (31)	
CAR product, *n* (%)					**0.001**
Axi‐cel	86 (51)	20 (53)	63 (62)	2 (15)	
Brexu‐cel	10 (6)	4 (11)	5 (5)	0	
Liso‐cel	12 (7)	1 (1)	7 (7)	2 (15)	
Tisa‐cel	18 (11)	4 (11)	3 (3)	5 (39)	
Ide‐cel	43 (25)	9 (24)	24 (23)	4 (31)	
Prior lines, median (range)	2 (1–10)	2 (1–5)	2 (1–10)	2 (1–7)	0.65
Prior auto‐HSCT, *n* (%)	55 (33)	11 (29)	32 (32)	5 (38)	0.82
Haemoglobin (g/dL)	11.1 (7.3–16.8)	11.3 (7.3–14.8)	10.75 (8–14.2)	11.1 (8.8–12.1)	0.41
Platelets (G/L)	196 (17–899)	189 (66–899)	199 (17–578)	230 (114–358)	0.87
ANC (G/L)	2.9 (0–33.6)	3.9 (1–33.6)	2.6 (0–15.4)	2.7 (1–7.4)	**0.013**
CRP (mg/L)	2.3 (0–204)	2 (0–101)	2 (0–204)	6 (0–16)	0.81
Ferritin (μg/L)	349 (11–6286)	319 (21–1428)	399 (11–6286)	317 (20–722)	0.27
LDH (UI/L)	211 (125–1232)	221 (125–662)	214 (130–1232)	192 (150–290)	0.13
CAR‐HEMATOTOX ≥2, *n* (%)	67 (40)	12 (32)	46 (45)	7 (54)	0.24
CRS, any grade, *n* (%)	153 (90)	36 (95)	95 (93)	11 (85)	0.40
CRS ≥3, *n* (%)	2 (1)	1 (3)	1 (1)	0	0.56
ICANS, any grade, *n* (%)	77 (46)	19 (50)	55 (54)	1 (8)	**0.007**
ICANS ≥3, *n* (%)	33 (20)	3 (8)	30 (29)	0	**0.002**
Tocilizumab, *n* (%)	96 (57)	19 (50)	68 (67)	5 (38)	0.050
Steroids, *n* (%)	90 (53)	20 (53)	64 (63)	3 (23)	**0.020**
ICU admission, *n* (%)	29 (17)	3 (8)	25 (24)	0	**0.012**

*Note*: Baseline laboratory values were determined before lymphodepleting chemotherapy with a leniency period of 3 days; values are median (range) unless otherwise specified. Categorical variables were compared by chi‐square or Fisher's exact test, continuous variables by Kruskal–Wallis test. *p*‐values <0.05 are in bold.

Abbreviations: ANC, absolute neutrophil count; axi‐cel, axicabtagene ciloleucel; brexu‐cel, brexucabtagene autoleucel; CRP, C‐reactive protein; CRS, cytokine release syndrome; DLBCL, diffuse large B‐cell lymphoma; FL, follicular lymphoma; HSCT, haematopoietic stem cell transplant; ide‐cel, idecabtagene vicleucel; ICAHT, immune effector cell‐associated haematotoxicity; ICANS, immune effector cell‐associated neurotoxicity syndrome; ICU, intensive care unit; LDH, lactate dehydrogenase; MCL, mantle cell lymphoma; PS, performance status; liso‐cel, lisocabtagene maraleucel; tisa‐cel, tisagenlecleucel.

Any‐grade ICAHT was observed in 153/169 patients (91.6%); severe (grade ≥3) ICAHT in 30%. We found no difference between lymphoma and MM patients (Table S1). Three subgroups emerged (Figure S2): isolated early ICAHT (*n* = 38, 24.8%), combined early and late ICAHT (*n* = 102, 66.7%) and isolated late ICAHT (*n* = 13, 8.5%). A clear gradient linked early and late severity (Figure 1): progression to grade 3–4 late ICAHT occurred in 13.8% of patients with no early ICAHT, 18.6% with grade 1, 33.3% with grade 2, 78.6% with grade 3 and 55.6% with grade 4. Overall, 69.6% of patients with severe early ICAHT progressed to severe late ICAHT, whereas 80.8% of those with grade 0–2 early ICAHT did not develop severe late events (Figure 1, Table S2). Among non‐severe ICAHT, isolated early (46.2%) or combined (42.4%) patterns predominated (Figure S2B); conversely, the majority of severe late ICAHT (54.9%) occurred independently of severe early ICAHT (Figure S2C). Median ANC kinetics followed a biphasic pattern in the combined group, whereas late‐only patients exhibited a delayed dip around week 5 (Figures S3 and S4); most patients, including those with severe ICAHT, recovered by day 60 (Figure S5).

**FIGURE 1:**
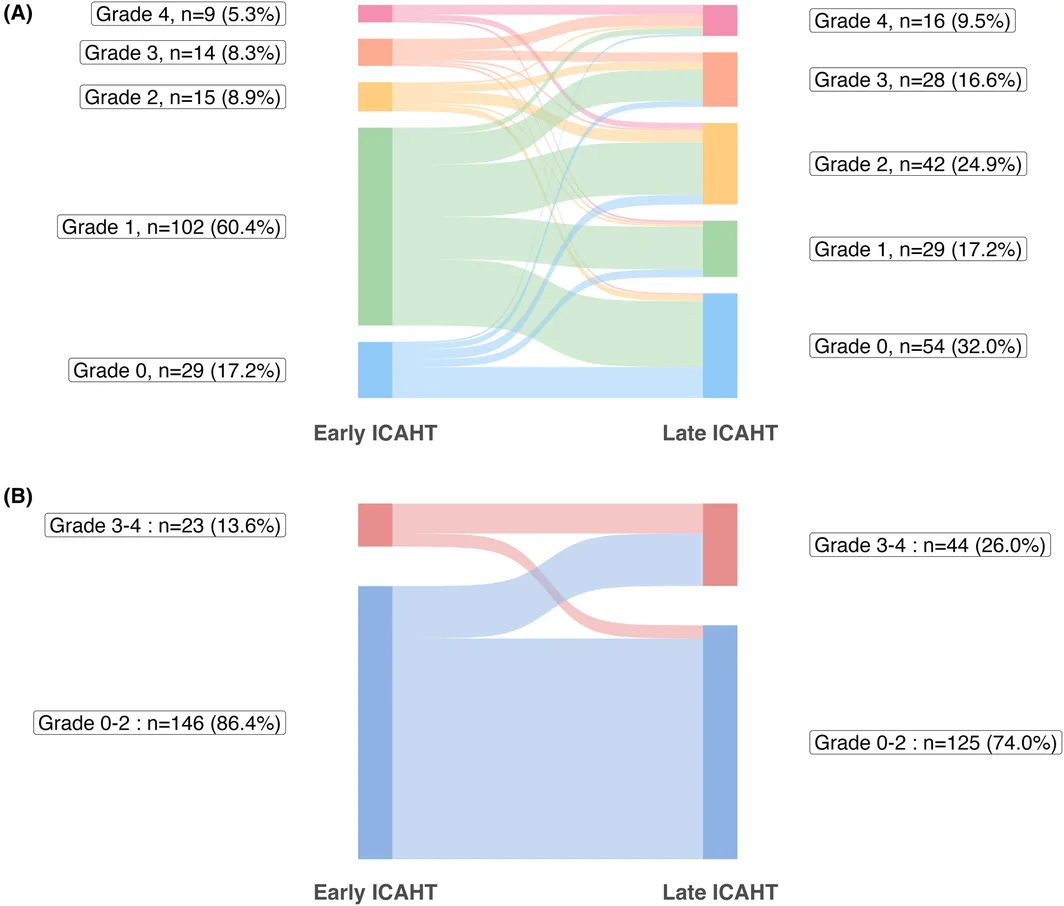
Transition from early to late immune effector cell‐associated haematotoxicity (ICAHT) (*n* = 169). (A) all grades; (B) non‐severe ICAHT (grade 0‐2) versus severe ICAHT (grade 3‐4). [Colour figure can be viewed at wileyonlinelibrary.com]

Each subgroup displayed distinct clinical features. The isolated early ICAHT group was predominantly male (84%, *p* < 0.05) and enriched in MCL (11% vs. 6% in the whole cohort). The combined group was enriched in DLBCL (64%). Strikingly, the isolated late ICAHT group was characterized by an overrepresentation of FL (31% vs. 10%), preferential use of tisagenlecleucel (tisa‐cel) (39% vs. 11%, *p* < 0.05) and ≥4 prior treatment lines in 46% of patients. This group also showed lower rates of any‐grade ICANS (8% vs. 46%, *p* < 0.05), no severe CRS or ICANS and reduced exposure to tocilizumab (38% vs. 57%, *p* < 0.05) and corticosteroids (23% vs. 53%, *p* = 0.05).

In this real‐world cohort, ICAHT affected over 90% of CAR T‐cell recipients, with 30% experiencing severe events—consistent with incidences reported by Liang et al^6^ and Rejeski et al.^1^, ^2^, ^3^ Our central finding is a strong continuum between early and late ICAHT: severe early events predicted severe late events in approximately 70% of cases, while preserved early counts largely protected against severe late cytopenias. This supports the hypothesis of shared biological drivers, including baseline marrow reserve, inflammatory milieu and CAR T‐cell expansion kinetics. The CAR‐HEMATOTOX score^1^—designed to capture early haematotoxicity—performed less well for late events in our data (high score in 72% of severe early vs. only 53% of severe late ICAHT), and complementary tools such as the Endothelial Activation and Stress Index (EASIX)‐based stratification proposed by Frenking et al^8^ or the KyoTox A‐score^9^ suggest that integrating endothelial and metabolic markers may improve late risk prediction. Late grade 4 ICAHT presented as persistent neutropenia without recovery, consistent with a continuum of the aplastic phenotype and heightened infection risk,^10^ grade 3 late events more often displayed intermittent neutropenia presumably modulated by G‐CSF and potentially manageable in outpatient care.

A clinically relevant subset (8.5%) experienced isolated late ICAHT without preceding early events. This group was enriched in FL, tisa‐cel exposure and heavily pre‐treated patients, with low rates of CRS/ICANS and reduced immunosuppressant use—a phenotype suggesting distinct pathophysiology, possibly involving more gradual immune activation or product‐specific effects. Whether tisa‐cel intrinsically predisposes to delayed cytopenias or whether the FL substrate is the primary driver cannot be disentangled here, and the contribution of CAR construct design to late ICAHT remains debated.^11^, ^12^ While the small number of patients in this subgroup limits analyses, we highlight this finding as hypothesis‐generating and call for dedicated prospective studies in this population.

Tocilizumab, which can induce neutropenia in rheumatological settings,^13^ was not differentially used across groups.

Limitations of this study include the retrospective design, the exclusion of patients dying or progressing early (likely biasing against high‐inflammation phenotypes), the lack of data regarding ciltacabtagene autoleucel (cilta‐cel), widely used now for MM but not available in France during the study period, and the absence of routine bone marrow assessments and incomplete granular data on G‐CSF refractoriness. Strengths include a relatively large unselected cohort, longitudinal ANC data over 90 days and the application of the EHA/EBMT consensus and validated algorithms.

Overall, our results identify early ICAHT as a strong, actionable predictor of late ICAHT and define a distinct late‐only population warranting dedicated study; integrating early haematological trajectories into late‐risk frameworks could refine surveillance and supportive care after CAR T‐cell therapy.

## AUTHOR CONTRIBUTIONS

**Roch Houot:** Conceptualization; methodology; validation; writing – review and editing; supervision; investigation. **Faustine Lhomme:** Investigation. **Mana Alshehri:** Methodology; software; investigation. **Samuel Vic:** Conceptualization; investigation; methodology; validation; writing – review and editing; supervision. **Malvina Bichon:** Conceptualization; writing – original draft; methodology; writing – review and editing; software; data curation; investigation. **Guillaume Manson:** Investigation.

## FUNDING INFORMATION

No funding was received for this research.

## CONFLICT OF INTEREST STATEMENT

R.H. reports honoraria from Kite/Gilead, Novartis, Incyte, Janssen, MSD, Takeda and Roche and consultancy for Kite/Gilead, Novartis, Bristol‐Myers Squibb/Celgene, ADC Therapeutics, Incyte and Miltenyi. G.M. reports honoraria from Kite/Gilead, Novartis, Takeda, Roche, BMS, AstraZeneca and CSL Behring. F.L. reports honoraria from Kite/Gilead, Novartis, Janssen, Amgen and Beone. The remaining authors declare no competing financial interests.

## Supporting information


**Table S1.** ICAHT distribution by disease sub‐groups (*n* = 169).

**Table S2.** Cross‐distribution of early and late ICAHT grades (*n* = 169). Values are n (%) within each early ICAHT grade (row‐wise percentages). Among patients with severe (grade 3–4) early ICAHT, 16 of 23 (69.6%) developed severe (grade 3–4) late ICAHT. Conversely, of 146 patients with grade 0–2 early ICAHT, only 28 (19.2%) developed severe late ICAHT.

**Figure S1.** Flow chart of patients included in the study. The abbreviation CAR denotes chimeric antigen receptor, ALL acute lymphoid leukaemia.

**Figure S2.** Distribution of early and late ICAHT—Venn diagrams. (A) All patients with any grade ICAHT (*n* = 153): isolated early (24.8%), combined early and late (66.7%) and isolated late ICAHT (8.5%). (B) Non‐severe ICAHT, grades 1–2 (*n* = 132): isolated early (46.2%), combined (42.4%) and isolated late (11.4%). (C) Severe ICAT, grades 3–4 (*n* = 51): isolated early (13.7%), combined (31.4%) and isolated late (54.9%).

**Figure S3.** Aggregated absolute neutrophil count (ANC) over time in the whole cohort (*n* = 169). Solid line, median ANC; shaded ribbon, interquartile range (IQR). The dotted horizontal line represents the ANC threshold of 500 cells/μL.

**Figure S4.** ANC evolution stratified by ICAHT severity (*n* = 169). Non‐severe ICAHT, blue; severe ICAHT, red. The dotted horizontal line represents the ANC threshold of 500 cells/μl.

**Figure S5.** ANC evolution in patients with late ICAHT, by grade. Solid lines represent median ANC; shaded ribbons represent the interquartile range (IQR). The dotted horizontal line represents the ANC threshold of 500 cells/μL.

## Data Availability

The data that support the findings of this study are available on request from the corresponding author. The data are not publicly available due to privacy or ethical restrictions.
