# Supplementary material for: Temporal dynamics of immune effector cell‐associated haematotoxicity: Linking early and late cytopenias after chimeric antigen receptor T cells
Source: Br J Haematol. 2026 Jul 10;209(3):1399–403. doi: 10.1111/bjh.70659 (PMC13570221; doi:10.1111/bjh.70659)
Supplement: Supplementary file 1 [file BJH-209-1399-s001.docx]

**Table S1. ICAHT distribution by disease sub-groups (n = 169).**

**Early ICAHT**

| **Disease** | **Grade 0** | **Grade 1** | **Grade 2** | **Grade 3** | **Grade 4** |
| --- | --- | --- | --- | --- | --- |
| Lymphoma | 19 (15.1 %) | 74 (58.7 %) | 14 (11.1 %) | 11 (8.7 %) | 8 (6.3 %) |
| Multiple myeloma | 10 (23.3 %) | 28 (65.1 %) | 1 (2.3 %) | 3 (7.0 %) | 1 (2.3 %) |

**Late ICAHT**

| **Disease** | **Grade 0** | **Grade 1** | **Grade 2** | **Grade 3** | **Grade 4** |
| --- | --- | --- | --- | --- | --- |
| Lymphoma | 39 (31.0 %) | 19 (15.1 %) | 34 (27.0 %) | 19 (15.1 %) | 15 (11.9 %) |
| Multiple myeloma | 15 (34.9 %) | 10 (23.3 %) | 8 (18.6 %) | 9 (20.9 %) | 1 (2.3 %) |
