# Supplementary material for: Temporal dynamics of immune effector cell‐associated haematotoxicity: Linking early and late cytopenias after chimeric antigen receptor T cells
Source: Br J Haematol. 2026 Jul 10;209(3):1399–403. doi: 10.1111/bjh.70659 (PMC13570221; doi:10.1111/bjh.70659)
Supplement: Supplementary file 2 [file BJH-209-1399-s004.docx]

**Table S2. Cross-distribution of early and late ICAHT grades (n = 169).**

| **n (%)** | | **LATE ICAHT** | | | | |  |
| --- | --- | --- | --- | --- | --- | --- | --- |
|  |  | **Grade 0** | **Grade 1** | **Grade 2** | **Grade 3** | **Grade 4** | **Total** |
| **EARLY ICAHT** | **Grade 0** | 16 (55.2) | 4 (13.8) | 5 (17.2) | 3 (10.3) | 1 (3.4) | 29 (17.16) |
|  | **Grade 1** | 34 (33.3) | 22 (21.6) | 27 (26.5) | 16 (15.7) | 3 (2.9) | 102 (60.36) |
|  | **Grade 2** | 3 (20) | 1 (6.7) | 6 (60) | 4 (26.6) | 1 (6.7) | 15 (8.88) |
|  | **Grade 3** | 1 (7.1) | 1 (7.1) | 1 (7.1) | 5 (35.7) | 6 (42.9) | 14 (8.28) |
|  | **Grade 4** | 0 (0.0) | 1 (11.1) | 3 (33.3) | 0 (0.0) | 5 (55.6) | 9 (5.33) |
|  | **Total** | 54 (31.95) | 29 (17.16) | 42 (24.85) | 28 (16.57) | 16 (9.47) | 169 (100) |

Values are n (%) within each early ICAHT grade (row-wise percentages). Among patients with severe (grade 3–4) early ICAHT, 16 of 23 (69.6%) developed severe (grade 3–4) late ICAHT. Conversely, of 146 patients with grade 0–2 early ICAHT, only 28 (19.2%) developed severe late ICAHT.
